# Cytocompatible and osteoconductive silicon oxycarbide glass scaffolds 3D printed by DLP: a potential material for bone tissue regeneration

**DOI:** 10.3389/fbioe.2023.1297327

**Published:** 2024-01-04

**Authors:** Matheus Versão Carnieri, Daniele de Freitas Garcia, Rafael Voltolini, Neri Volpato, Marcio Mafra, Euclides Alexandre Bernardelli, Marco Augusto Stimamiglio, Carmen Kuniyoshi Rebelatto, Alejandro Correa, Lucas Freitas Berti, Bruna Hilzendeger Marcon

**Affiliations:** ^1^ Department of Mechanical Engineering, Postgraduate Program in Mechanical and Materials Engineering, Universidade Tecnológica Federal Do Parana, Curitiba, Brazil; ^2^ Laboratory of Basic Biology of Stem Cells (LABCET), Carlos Chagas Institute—FIOCRUZ-PR, Curitiba, Brazil; ^3^ Core for Cell Technology of Pontifical Catholic University of Paraná—PUCPR, Curitiba, Brazil; ^4^ Confocal and Eletronic Microscopy Facility (RPT07C), Carlos Chagas Institute—FIOCRUZ-PR, Curitiba, Brazil

**Keywords:** bone tissue engineering, bioactive glass, DLP 3D printing, scaffold, regenerative medicine, silicon oxycarbide glass, adult stem cell

## Abstract

Bone lesions affect individuals of different age groups, compromising their daily activities and potentially leading to prolonged morbidity. Over the years, new compositions and manufacturing technologies were developed to offer customized solutions to replace injured tissue and stimulate tissue regeneration. This work used digital light processing (DPL) technology for three-dimensional (3D) printing of porous structures using pre-ceramic polymer, followed by pyrolysis to obtain SiOC vitreous scaffolds. The SiOC scaffolds produced had an amorphous structure (compatible with glass) with an average porosity of 72.69% ± 0.99, an average hardness of 935.1 ± 71.0 HV, and an average maximum flexural stress of 7.8 ± 1.0 MPa, similar to cancellous bone tissue. The scaffolds were not cytotoxic and allowed adult stem cell adhesion, growth, and expansion. After treatment with osteoinductive medium, adult stem cells in the SiOC scaffolds differentiated to osteoblasts, assuming a tissue-like structure, with organization in multiple layers and production of a dense fibrous matrix rich in hydroxyapatite. The *in vitro* analyses supported the hypothesis that the SiOC scaffolds produced in this work were suitable for use as a bone substitute for treating critically sized lesions, with the potential to stimulate the gradual process of regeneration of the native tissue. The data obtained stimulate the continuity of studies with the SiOC scaffolds developed in this work, paving the way for evaluating safety and biological activity *in vivo*.

## 1 Introduction

The bone tissue is capable of self-regenerating after injury, recovering the structure and functionality of the original organ. However, when the damage reaches a critical size, complementary therapies are necessary for tissue regeneration (Al-Harbi et al., 2021). Currently, the gold standard treatment available is using bone grafts. Although this strategy has the potential to regenerate the injured tissue, it faces challenges such as pain at the collection site and the limited amount of material available when using autologous tissue. On the other hand, allografts face a reduction of biological response due to sample processing and the risk of transmission of pathogens. Furthermore, the implantation of grafts can cause inflammation and infection, compromising the success of the treatment (Brink, 2021).

To overcome these challenges, bone tissue engineering has explored biomaterials structured in three-dimensional (3D) scaffolds as a promising strategy for treating critical-sized bone defects. To substitute the damaged tissue and to promote regeneration, the 3D device must be non-cytotoxic, osteoconductive i.e., to allow the growth of bone tissue on its surface, and reflect the load-bearing properties of the original tissue. Bioglasses, also known as bioactive glasses, are non-crystalline ceramics that allow the formation of a hydroxyl carbonate apatite surface layer and effectively bond to the bone, promoting the formation of new bone tissue. These glasses are biocompatible, corrosion resistant, and may have bone-like mechanical properties (Al-Harbi et al., 2021).

The main component of silica-based bioactive glasses is silicon dioxide (Al-Harbi et al., 2021), which may be mixed with different compounds and pyrolyzed to produce scaffolds with specific features. Silicon is a tetravalent metalloid with high oxygen affinity, mainly found in its oxidized form as silica/silicate minerals (Götz et al., 2019). In the human body, it corresponds to approximately 0.01% of the mass, being found predominantly in connective and rigid tissue, such as bone (Götz et al., 2019). It positively impacts bone tissue homeostasis, with studies indicating a positive correlation between silicon consumption (in different forms) and bone tissue health and collagen production (Jugdaohsingh et al., 2004; Spector et al., 2008; MacDonald et al., 2012).

Silicon stimulates bone formation, either by promoting the secretion of factors, the osteogenic differentiation, and the mineralization process (Chen D. et al., 2022; Chen et al., 2022 X.; Ge et al., 2022; Yunsheng et al., 2023) or by regulating the osteoclastogenic activity (Schröder et al., 2012; Magnusson et al., 2021). Since the development of bioactive glass 45S5 by Hench in the 1960s, different compositions of silicon-based glasses have been developed and evaluated for applications in bone regeneration. An important feature of bioactive glasses is their ability to bond to bone and soft tissues. Although they do not reproduce the complexity of the native tissue, these glasses may be customized to deliver pharmaceutical compounds, such as antibiotics and anti-inflammatory elements (Al-Harbi et al., 2021), and to control inflammation and infection, which frequently compromise the success of bone grafts. Furthermore, biomaterials can be shaped and structurally customized to meet the patient’s needs (Al-Harbi et al., 2021; Marsh et al., 2021).

More recently, another composition that has been evaluated is bioactive glasses containing silicon oxycarbide (SiOC). SiOC glasses may have higher elastic modulus, bending strength, hardness, and chemical durability than silicate glasses (Lagonegro et al., 2017) and are also hemocompatible (Zhuo et al., 2005). SiOC nanowires produced by carbon doping of Si substrates did not impair the activation of platelets and enabled the adhesion, survival, and growth of murine fibroblasts (Lagonegro et al., 2017). SiOC(N) ceramic scaffolds may also be obtained from 3D thermoplastic polyurethane scaffolds produced by fused filament fabrication, impregnated with pre-ceramic polymer polysilazane and pyrolyzed. The scaffolds produced using this methodology were non-cytotoxic (tested with 3T3 murine fibroblasts) and supported cell adhesion and growth of MG63 cells derived from human osteosarcoma (Kulkarni et al., 2021). The same methodology was used to produce porous scaffolds (diameter = 13 mm; heigh = 1.5 mm; porous = 300–500 µm), which supported the adhesion, survival, and osteogenic differentiation of bone marrow-derived stem cells (Yang et al., 2021).

Our work aims to further explore the potential of SiOC scaffolds as pro-regenerative bone substitutes. The scaffolds designed were geometrically composed of triply periodic minimal surface (TPMS) diamond unitary cells packed to obtain pore chains in ordered cell deposition zones and achieve a good relationship between porosity and mechanical properties. The TPMS structures are continuous and periodic surfaces with crystallographic group symmetries, free from self-intersections, and they hold significance in applications involving topological complexity (Abueidda et al., 2019). From a manufacturing perspective, in the context of additive manufacturing technologies, the mentioned characteristics of TPMS structures prevent the formation of floating points between layers, thereby reducing the likelihood of geometric printing failures. These structures are well-suited for mass transfer application due to their continuity and less tortuous pores, resulting in significantly improved structure permeability (Yeranee and Rao, 2022).

To obtain the SiOC with this complex design, we used pre-ceramic polymers and digital light processing (DLP) technique followed by thermal treatment. In the DLP, a projector hardens an entire slice of the 3D structure, speeding up the manufacturing process. The DLP system also uses a bottom-up printing process, where the printed layers are shifted up and are no longer immersed in the slurry, reducing the risk of inserting defects in the printing process (Chen et al., 2019). The photocured sample is submitted to thermal treatment in an inert atmosphere (pyrolysis), where heating first leads to the crosslinking of the pre-ceramic polymer and then to the loss of the organic moieties (debinding) and shrinkage, producing a ceramic matrix (Colombo et al., 2010; Schmidt and Colombo, 2018). Although the DLP technique with pre-ceramic polymers has already been described to produce SiOC parts, to the best of our knowledge, this is the first time that this system has been used to manufacture cytocompatible 3D SiOC scaffolds for use in bone tissue engineering.

## 2 Materials and methods

### 2.1 Preparation of PDC resins

**FIGURE 1:**
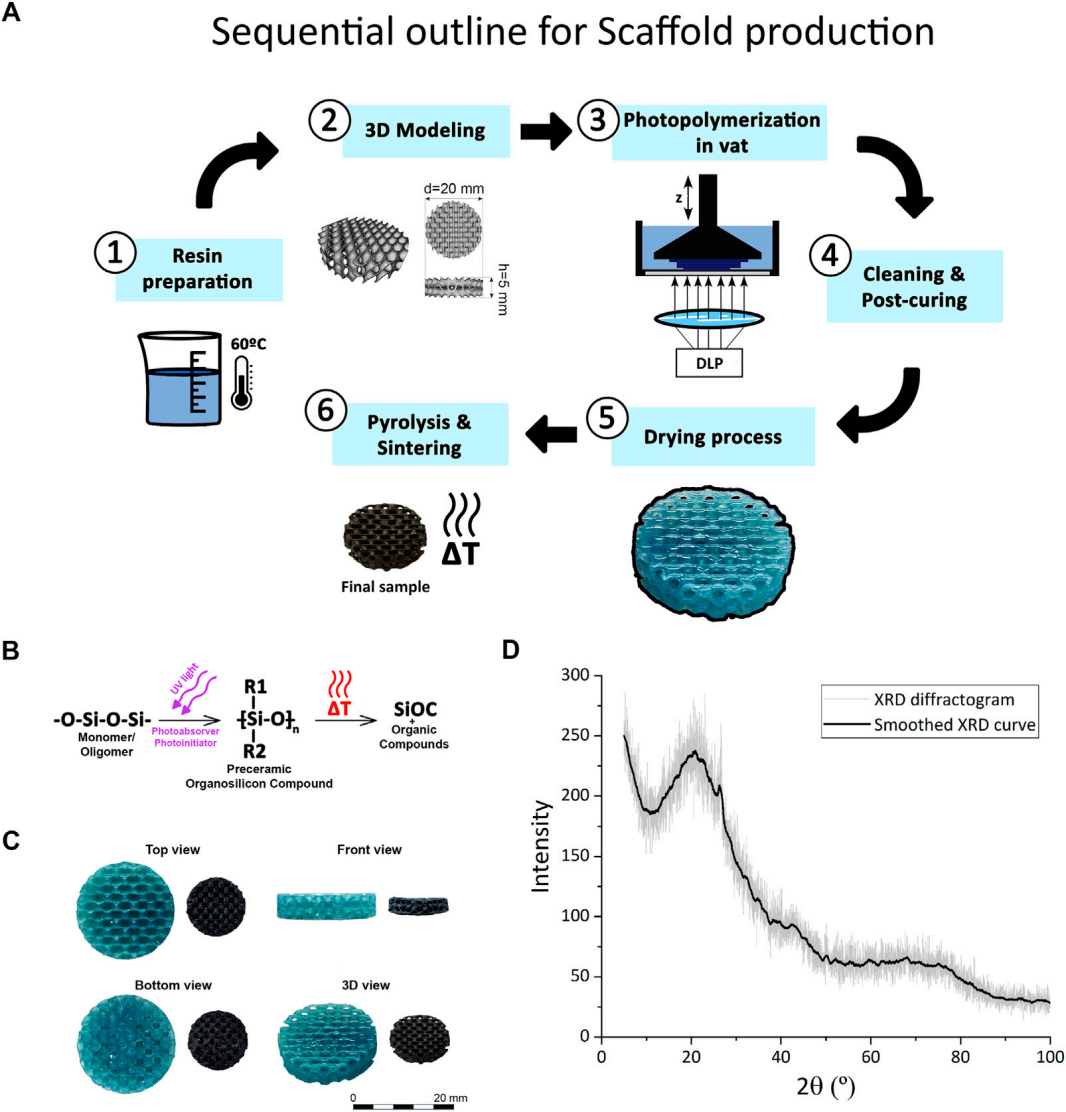
SiOC scaffold production. **(A)** Workflow of SiOC scaffold production. **(B)** Obtaining vitreous SiOC from pre-ceramic polymer. **(C)** Top, front, bottom, and 3D views of scaffolds pre (left) and pos-pyrolysis (right). **(C)** Mass variation between the printing and drying stages and between drying and pyrolysis stages. **(D)** Diffractogram of the final material obtained after pyrolysis.

The workflow for producing SiOC scaffolds is described in Figure 1A. The resin used for the scaffold manufacturing project comprises a mixture of pre-ceramic polymer, ceramic powder fillers, photoinitiators/photoabsorbers, and a solvent (Schmidt and Colombo, 2018). TEGO RC 711 siloxane (Parafix LTDA and Vonka LTDA) was the chosen pre-ceramic polymer, and it acted as the base of the photopolymerizable system when combined with the photoinitiator. Silres H44 powder (Polisil Silicones), a phenylmethyl polysiloxane, served as the silica ceramic filler. It demonstrated good solubility in various solvents, quick curing in the presence of catalysts, and excellent thermal stability. Toluene (Dsyslab) acted as the solvent due to its availability and compatibility with H44. The photoinitiator, Irgacure 819 (IGM Resins), and the photoabsorber, Erioglaucine A (Brilliant Blue FCF) (Sigma Aldrich), were used to facilitate the photopolymerization process.

To prepare the resin, Silres H44 was mixed with toluene in a 1:1 weight ratio using a Fisherbrand magnetic stirrer at 60°C for 2 h. Then, TEGO RC 711 was added in the same weight amount as Silres H44. Omnirad 819, the photoinitiator, was added at a 2% weight ratio relative to RC711, while Erioglaucine A (E133), the light photoabsorber, was added at a 0.75% ratio relative to the H44 polysiloxane.

### 2.2 3D modeling of scaffolds

The project’s definition of the sample geometry had four main concepts: controlled pore size, ordered macropore structure, suitability for 3D printing, and adaptation to the containers used in biological testing (cylindrical and small containers). Therefore, the modeled geometry for the project followed a discoidal shape with diamond TPMS (Triply Periodic Minimal Surface) cells. The parts were designed using the advanced lattice design software nTopology.

### 2.3 Photopolymerization-based 3D printing, debinding, and pyrolysis

The ceramic scaffolds were produced using an ANYCUBIC 3D printer, model Photon Ultra, with DLP (Digital Light Processing) technology (SLIM 3D Printers). It operates in the visible light range with a UV (Ultraviolet) light source and a wavelength of 405 nm. The exposure time was 10 s, with 15 s for the base layers, and the layer height was set to 100 µm. Eight pieces were printed per process for the cylindrical scaffolds, resulting in a total print time of 23 min. Meanwhile, two samples were obtained per process for the quadrangular scaffolds (used in the three-point bending tests), with a total print time of 43 min.

After the 3D printing, the parts were removed from the printing platform and transferred to the Anycubic Wash and Cure 2.0. The machine was set to its cleaning function and runned for 5 min to remove any residual uncured resin from the macropores and the surface of the scaffolds. Subsequently, the samples were post-cured for 1 min using the same equipment, which utilizes a set of UV lamps to complete the curing process initiated during the photopolymerization in the tank.

Next, the cured structures were dried in a sterilization and drying oven (ETHIKTECHNOLOGY) at 60°C for 24 h. This step removed any traces of cleaning solvent from the surfaces. It completed the preparation cycle of the pre-ceramic samples, resulting in dry parts without any accumulated excess resin on the surfaces.

Finally, completing the manufacturing process, the samples proceed to the thermal debinding step through pyrolysis (Figure 1B). The heating rates were 0.5°C/min for the crosslinking and decomposition zones and 1.5°C/min and 5°C/min for the other zones, with waiting times of 60 min at 380°C, 500°C and 1000°C, as described in Table 1.

**TABLE 1:** Heating rate sequence for the pyrolysis process.

Step	Temperature (°C)	Heating rate (°C.min^-1^)	Waiting times (min)
1	25 up to 280	1,5	0
2	280 up to 380	0,5	0
3	380 up to 500	0,5	60
4	500 up to 650	0,5	60
5	650 up to 1000	5	0
6	1000 up to 25	5	60

### 2.4 Material and mechanical characterization

#### 2.4.1 Phase composition by X-ray diffraction test

For X-ray diffraction (XRD), the samples were ground in a high-resistant porcelain mortar to obtain a fine homogeneous powder. In total, 15 g of powder material was used for the assessment. Subsequently, the analysis was conducted using a Shimadzu XRD-7000 X-ray diffractometer with an adjustable radius (200–275 mm) and a power of up to 3 kW. Regarding the measurement conditions, a Copper (Cu) target was used, with a regular focus condition, a power of 2 kW, a voltage of 30 kV, and a current of 30 mA. A continuous scanning mode was applied, with a measurement range varying from 5 to 100° and a step size of 0.02° every 0.6 s.

#### 2.4.2 Mass variation

To determine the mass variation, fifty-five cylindrical scaffolds were manufactured, following the geometry standards outlined in Section 2.2. All samples were weighed using an ECLIPSE analytical balance, model EBL2141, with a resolution of 0.0001 g, after the printing, drying, and pyrolysis steps. Subsequently, the percentage mass variation throughout the processes was calculated using Eq. 1, providing an indicative factor for the volume shrinkage of the parts.

Equation 1 Calculation of mass variation between scaffolds before and after pyrolysis.

$$Mass\;variation=\left(\left(mbp-map\right)/map\right).100$$ (1)

In the equation, “mbp” represents the mass of the samples before pyrolysis, and “map” represents the mass of the samples after pyrolysis.

#### 2.4.3 Density, porosity, and size of macropores

Firstly, the density of the material was determined using the helium picnometry technique, following the ASTM D6226-21 and ASTM D2638-06 standards (ASTM Standard D2638-21, 2021; ASTM-D6226-21, 2021). The test was conducted using a Quantachrome MVP-D160-E Helium Multipycnometer. Analytical helium gas was at 20 psi, with an operating temperature of 22°C, a maximum cell diameter of 50 mm, and a maximum pressure reading of 20 psi. In total, 15 g of SiOC powder (obtained through the process outlined in Section 2.4.1) was utilized.

The total volume of the scaffolds (Vs.) was calculated using Eq. 2, where “ma” is the average mass measured for the fifty-five pyrolyzed scaffolds and "ρ" is the material density in g/cm³. Then, the volume of a solid cylindrical sample (Vc) was calculated using the average values of diameter (dm) and height (hm) measured manually after pyrolysis (Eq. 3) using a VONDER analog universal caliper.

Equation 2 Determination of the total volume of the scaffolds (Vts).

Vs=ms/ρ (2)

Equation 3 Determination of the total volume of a solid cylindrical sample after pyrolysis.

$$Vc=\left(\pi .dm^{2}.hm\right)/4$$ (3)

Finally, with the knowledge of the total volume of porous scaffolds (Vs.) and the total volume of a solid cylinder (Vc), the theoretical porosity after pyrolysis can be determined using Eq. 4.

Eq. 4 Calculation of porosity.

$$Porosity\;\left(\%\right)=100.\left(\left(Vc-Vs\right)/Vc\right)$$ (4)

Furthermore, the size of macropores was also determined using Scanning Electron Microscopy (SEM), with the measurement of lengths performed using the measurement tools provided by the ImageJ image analysis software.

For comparison with the manufactured scaffolds, the porosity and theoretical macropore diameter (before pyrolysis) of the designed 3D models were directly provided by the Ntopology software, using the option “*mass properties from body/mesh*” for a specific reference volume.

#### 2.4.4 Fourier transfom infrared (FT-IR) spectroscopy

Fourier transform infrared (FT-IR) spectroscopy (Bruker, IFS-120HR/FRA-106S) was carried out in the absorbance mode to characterize the chemical bonds in the SiOC scaffold, which was previously milled to make KBr disc with the SiOC powder. The chemical bonds were evaluated using FT-IR, recorded between 400 and 4000 cm^−1^ wavenumber with a resolution of 2 cm^−1^, and 4 scans.

#### 2.4.5 Thermogravimetric analysis (TGA)

The thermal stability of the SiOC samples after pyrolysis was investigated using TGA-51H Thermogravimetric Analyzer (Shimadzu) using a platinum cell from room temperature to 1200°C at a heating rate of 10°C/min and a nitrogen flow of 50 mL/min.

#### 2.4.6 Ion release analysis

For ion release analysis, SiOC scaffolds (1800 mg) were submerged in 180 mL of 0.05 M Tris-HCl buffer (pH 7.3) and kept in an incubator at 37°C for 15 days. After 1, 5, 10, and 15 days, 5 mL of solution was collected and replaced by fresh Tris-HCl buffer. As blank control, Tris-HCl buffer was incubated in the same conditions, without SiOC samples.

A standard stock solution containing 1000 μg/mL Si from AccuStandard (New Haven, United States of America) was used to prepare external calibration solution. The samples were analyzed using an VISTA PRO ion-coupled plasma employing optical emission spectrometer (ICP-OES) (Varian, Mulgrave, Australia). Peak height intensities were measured at the 288.158 nm Si(I) emission line. Each sample was measured three times (technical replicates). The concentration of ions released at each time corresponds to the measurement obtained minus the average blank value.

#### 2.4.7 Microhardness

First, one cylindrical sample with a diameter of d = 20 mm and constant volume was pyrolyzed under the conditions described in Section 2.3. The specimen was then pre-prepared by embedding it in bakelite using a Struers Predopress embedding machine. The sample was placed inside the equipment and subjected to a preheating time of 5 min, a heating time of 10 min, a cooling time of 5 min, and an applied force of 30 kN. Once embedded, the sample was prepared using a Struers Knuth-Rotor grinder, following the following sequence of grit sizes: 220, 320, 400, 600, and 1200.

The sample was polished by applying cyclic rotational movements on a polishing cloth with alumina for 2–3 min, using an Arotec APL-4 metallographic polisher with medium particle size alumina suspension (1 µm).

The specimen was placed in an EMCO-TEST M4C 025G 3M universal hardness testing machine with a load force range of 1–50 kgf. Due to the ceramic sample’s inherent hardness, multiple indentations were made across the specimen at a magnification of ×147, using a load of 1 kgf for 10 s. The diamond-shaped indentations were identified and measured using the ECOS software provided by the EMCO-TEST machine. The Vickers hardness (HV) was calculated using the following formulas: Eq. 5.

Equation 5 Calculation of vickers hardness (HV)

$$HV=\left(1,844.F\right)/dm^{2},dm=\left(d1+d2\right)/2$$ (5)

Where “F" (kgf) is the applied load force, “dm” (µm) is the average diagonal length, and “d1″ and “d2” (µm) are the main diagonals of the indentation.

#### 2.4.8 Three-point flexural test

For the three-point flexural tests, a new quadrangular and elongated geometry was modeled in nTopology, with a length of 95 mm, width of 15 mm, and height of 8 mm. The samples followed the same filling pattern of diamond TPMS cells. Eight samples were produced and characterized based on flexural modulus, following the ASTM C1161-18 (2023) standard for three-point bending tests (ASTM-C1161-18, 2023).

The tests were conducted on the EMIC DL10000 universal testing machine using the following settings: a maximum applied force (F) of 50 kgf on the samples, a support point distance (L) of 40 mm, and a measurement speed (s) of 1 mm/min. Finally, the flexural strength of the samples was calculated using the TESC software, based on the following Eq. 6.

Equation 6 Calculation of flexural strength.

$$S=\left(3.P.L\right)/\left(2.b.d^{2}\right)$$ (6)

Where “S" is the flexural strength, “P" is the applied rupture force, “L" is the outer support span, “b" is the width of the sample, and “d" is the thickness of the sample at the measured central point.

### 2.5 Biological characterization

#### 2.5.1 hASCs isolation and culture

The human adipose tissue-derived stromal cells (hASCs) were isolated from lipoaspirated adipose tissue from donors, as previously described by the group and with the approval of the Ethics Committee of Fundação Oswaldo Cruz, Brazil (CAAE number: 48374715.8.0000.5248). For isolation, 100 mL of adipose tissue was washed with sterile phosphate-buffered saline (PBS) (Gibco Invitrogen). One-step digestion with 1 mg/mL type I collagenase (Gibco Invitrogen) was performed (30 min; 37°C; permanent shaking), followed by filtration through 100 μm, then 40 μm mesh filter (BD Biosciences). The cell suspension was centrifuged (10 min at 800 G; 8°C), and incubated with erythrocyte lysis buffer pH 7.3 (5 min). After 24 h, non-adherent cells were removed, and the culture medium was changed twice a week (Rebelatto et al., 2008; Marcon et al., 2020).

The hASCs were cultured in DMEM medium (Dulbecco’s modified Eagle’s, Sigma, D5523) supplemented with 10% fetal bovine serum, L-Glutamine 2 mM, penicillin (100 U/mL) and streptomycin (100 μg/mL) at 37°C and 5% CO_2_ atmosphere.

#### 2.5.2 Cell seeding on the scaffolds

SiOC scaffolds were washed three times with isopropanol for 1 hour, dried at 60°C, and sterilized for 2 hours in an incubator at 160°C. hASCs were maintained in culture in polystyrene culture flasks until 80% confluence, with medium changes every 2–3 days. Then they were detached using a 0.05% trypsin, 0.02% EDTA solution and centrifugated for 5 min at 700 G. The cell pellet was suspended in new medium, and the concentration of 1 × 10^5^ cell/scaffold was seeded onto the scaffolds following the protocol described by Fairag et al. (2019) and Biagini et al. (2021) (Fairag et al., 2019; Biagini et al., 2021). Briefly, two scaffolds were placed in a syringe barrel connected to a four-way stopcock. The cell suspension was added, the syringe plunger was inserted, and the syringe flipped slowly. With one of the stopcocks open, the syringe plunger was pushed, securing the scaffolds within the cell’s suspension, then the four-way stopcock was closed again. The syringe was placed in a humid incubator (37°C and 5% CO_2_) and turned 90° every 20 min for 2 hours and 40 min. The scaffolds were placed in a 24-well ultra-low attachment plate with 1 mL of supplemented culture medium and kept at 37°C and 5% CO_2_.

#### 2.5.3 Direct contact assay

To cytotoxicity evaluation, hASCs were plated in 6-well plates with 2.5.10^5^ cells/well and cultured in supplemented DMEM medium for 24 h in an incubator at 37°C and 5% CO_2_. The next day, the medium was changed, and scaffolds were positioned on the top of the cells in the center of each well. SDS (Sodium dodecyl sulfate, Sigma) was solubilized at 200 μg/mL in DMEM medium as a positive control of cell death. The negative control of the assay was the cells in the supplemented DMEM medium. After 48 h, cell morphology was analyzed under an inverted microscope (Leica DMIL LED), and ten images of each well were acquired. For each condition, three technical replicates were performed.

#### 2.5.4 Growth curve

For the growth curve analysis, the cells seeded on the scaffolds were detached using 0.05% trypsin-EDTA, and the number of cells was counted in Neubauer’s chamber. The analysis was performed immediately after cell seeding on the scaffolds (t = 0) and after 7 (t = 7d) and 14 days (t = 14d) of culture. Three technical replicates were performed. Fluorescence and scanning electron microscopy analysis were also performed for each time point.

#### 2.5.5 Immunofluorescence

The cells were fixed with 4% paraformaldehyde for 20 min. For fluorescence, cells were permeabilized with PBS/Triton 0.1% and incubated with anti-β-tubulin rabbit polyclonal antibody (Invitrogen, PA5-16863) and anti-rabbit IgG conjugated with Alexa 488 secondary antibody (Invitrogen, A27034). The 300 nM DAPI staining solution was used to dye the nucleus of the cells. The samples were analyzed under the DMI6000B fluorescence microscope (Leica) and the TCS SP5 confocal microscope (Leica).

#### 2.5.6 Scanning electron microscopy

For scanning electron microscopy analysis, the SiOC scaffolds (without cells) were first coated with gold (Leica EM ACE200) and then analyzed using a JSM6010 PLUS-LA (JEOL) scanning electron microscope.

The scaffolds containing cells were first fixed (2.5% glutaraldehyde; 0.1 M sodium cacodylate buffer) for 1 hour at room temperature and stored at 4°C (in the same solution) until used. The samples were then washed (0.1 M sodium cacodylate buffer), post-fixed (1% osmium tetroxide; 0.1 M sodium cacodylate buffer) for 40 min, and washed again. The samples were dehydrated with a series of ethanol solutions with increasing concentrations (30%, 50%, 70%, 90%, and 100%), dried using a critical point (Leica EM CPD300), and coated with gold (Leica EM ACE200). The samples were analyzed using a JSM6010 PLUS-LA (JEOL) scanning electron microscope. For energy-dispersive X-ray spectroscopy (EDS), the post-fixation step with osmium tetroxide was omitted, and the samples were coated using carbon.

#### 2.5.7 Colonization assay

One scaffold (without cells) was placed on a confluent hASC monolayer (in a six well culture plate). The migration of the cells from the plate to the scaffold was analyzed after 2 days. The nuclei of the cells were labeled with 300 nM DAPI staining solution, and the cytoskeleton with anti-β-tubulin antibody. Then, the scaffolds were analyzed under a DMI6000B fluorescence microscope (Leica).

#### 2.5.8 Immunophenotypic profiling

At 14°day of culture, the scaffolds were washed with BSS-CMF and incubated with 0.05% trypsin-EDTA to detach the cells. 8.10^5^ cells were blocked with PBS/BSA 0.5% for 1 hour and 30 min at 4°C. After that, the tubes with 2.10^5^ cells were centrifuged at 700 G for 5 min and resuspended in a 50 µL solution of PBS/BSA 1% with the antibodies, incubated for 1 hour at 4°C in the dark, then washed and resuspended in 300 µL of PBS 1X. The antibodies used were anti-CD90—FITC (Invitrogen, 11-0909-42); anti-CD105—PE (eBiocience, 12-1057-73); anti-CD73—APC (eBiocience, 17-0739-42); anti-CD34—FITC (Invitrogen, 11-0349-42); anti-CD11b—PE (Invitrogen, 12-0112-82), anti-CD45—APC (eBiociences, 17-0459-73), anti-CD19—FITC (BD Pharmigen, 555412); anti-HLA-DR—APC (Life Technologies, MHLDH05); and the isotype controls IgG—FITC (BD Pharmigen, 555748); IgG—PE (BD Pharmigen, 555749); IgG—APC (BD Pharmigen, 555751). The experiment was performed once.

Cells grown in polystyrene culture flasks and prepared similarly were used for comparison. Samples were acquired with the FACSCanto II Flow Cytometer (BD Biociences) with 100000 events per tube. Data were analyzed with FlowJo software version 10.9.0.

#### 2.5.9 Evaluation of osteogenic differentiation

The hASCs were seeded on the SiOC scaffolds and cultivated for 7 days. Then, the culture medium was changed to Mesenchymal Stem Cell Osteogenic Differentiation Medium (Lonza), with medium changes every 2–3 days. After 21 days of treatment with an induction medium, the scaffolds were fixed for fluorescence and scanning electron microscopy analysis. To identify hydroxyapatite deposition, the scaffolds were incubated with the OsteoImage Kit (Lonza, PA-1503) according to the manufacturer’s instructions and analyzed under DMI6000B fluorescence microscope (Leica). Three technical replicates were performed.

## 3 Results

### 3.1 Development of porous SiOC scaffolds produced by DLP and pyrolysis

After printing by DLP, green samples were dried and subsequently pyrolyzed (Figure 1C). Overall, the most significant mass variations occurred between the drying and pyrolysis stages, with an average decrease of 71.8% ± 1.9% for the 55 measured samples. In contrast, there was an average decrease of 12.7% ± 4.8% between the printing, cleaning, and drying stages.

XRD analysis highlighted the relationship between the diffraction angle 2θ and the quantity of diffracted X-rays for each angle (I) (Figure 1D). The established diffraction pattern does not exhibit sharp, well-defined peaks but appears continuous. These characteristics indicate that the studied sample possesses an amorphous structure. Additionally, two curvatures can be observed in the graph: the first around the angle of 2θ = 22°, which is more predominant, and the second around 2θ = 40°. These characteristics correspond to diffraction patterns of amorphous silica and graphite carbon. Therefore, based on the diffractogram, it can be concluded that the studied ceramic material lacks a crystalline structure and can be considered vitreous.

It was necessary to determine the density of the final material to determine the porosity of the manufactured and pyrolyzed scaffolds. The Helium pycnometer test yielded an average density of 1.92 ± 0.02 g/cm³. The average mass of the produced scaffolds, measured on a precision balance, was 0.2048 g ± 0.017. By combining the mass with the known density, the calculated total volume of the scaffolds was Vts = 106.67 ± 9.97 mm³.

The average values of diameter d = 12.72 ± 0.41 mm and height h = 3.07 ± 0.20 mm of the produced pieces were used to determine the volume of the solid cylindrical geometry. Based on this, the estimated value for the volume of the solid geometry (Vt) was 390.12 ± 50,60 mm³. With the values of Vts and Vt after pyrolysis, the estimated final porosity for the pyrolyzed scaffolds was 72.69% ± 0.99%.

For the macropore size of non-pyrolyzed scaffolds, initially, a standard macropore size of 1.5 mm was assigned to the model designed via Ntopology. In the measured models, obtained through optical microscopy, an average diameter size of 1.43 ± 0.06 mm was observed. Subsequently, in the case of pyrolyzed scaffolds, measured using SEM and considering only the largest visible diameters of the macropores, an average value of 741.6 ± 39.6 µm was obtained. And finally, regarding the geometry and porosity values of the 3D models before the pyrolysis/sintering processes, the theoretical porosity and macropore size obtained via Ntopology were 76.7% and 1.5 mm, respectively.

**FIGURE 2:**
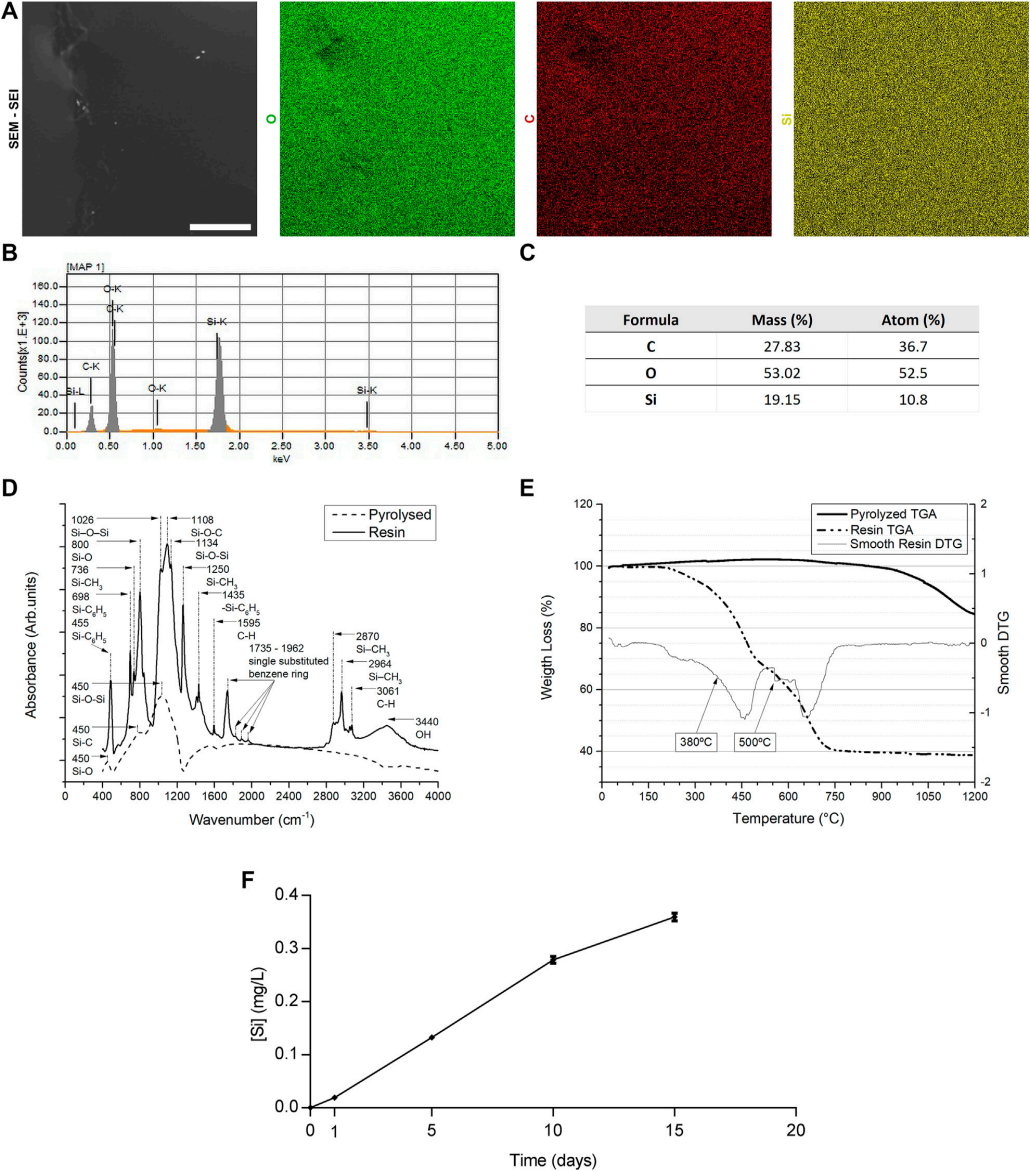
Chemical and physical characterization of SiOC scaffolds. EDS analysis of SiOC scaffold: **(A)** mapping, **(B)** spectrum and **(C)** table. **(D)** FT-IR spectra and **(E)** TGA analysis of SiOC scaffolds. **(F)** Si ion release analysis (n = 3; technical replicates).

Using EDS, we confirmed that the pyrolyzed scaffolds were composed of silicon, carbon, and oxygen (Figures 2A–C and Supplementary Figure S1), which were homogeneously distributed in the material (Figure 2A). By FT-IR analysis (Figure 2D), we obtained a spectra over a wavenumber range between 400 and 4000 cm^−1^ of the resin and SiOC (Pyrolyzed) scaffolds band. The characteristic band of the resin scaffold showed absorption bands due to Si-O-Si (around 1055 cm^−1^), Si-O-C (around 1105 cm^−1^), Si-CH3 (around 950 and 1250 cm^−1^), and -OH (3700–3800 cm^−1^), in addition to those of the Si substrate. The 1775–1962 cm^−1^ characteristic bands were associated with the single substituted benzene ring. TGA analysis for the pyrolyzed sample demonstrated that mass change is not observed until 900°C, but for temperatures higher than 1000°C this material begins to deteriorate (Figure 2E). For the resin sample, the weight loss started at 200°C, with two major mass chages at 380°C and 500°C (Figure 2E). We also monitored silicon ion release for 15 days. The concentration of ions released into the medium increased until day 15. However, from day 10 onwards, there was a reduction in the release rate (Figure 2F).

### 3.2 Mechanical characterization of SiOC scaffolds

For the solid cylindrical sample produced, the average lengths of the measured indentation diagonals, d1 and d2, were found to be 44.4 ± 2.8 µm and 44.8 ± 0.8 µm, respectively. The average hardness was determined to be 935.1 ± 71.0 HV.

The average maximum flexural stress for the tests was 7.8 ± 1.0 MPa. As for the dimensions of the samples, the width and height had an overall average of 10.5 ± 1.2 mm and 6.9 ± 1.0 mm, respectively. However, it is noticeable that the first three samples had larger dimensions than the others, which may influence this property.

**FIGURE 3:**
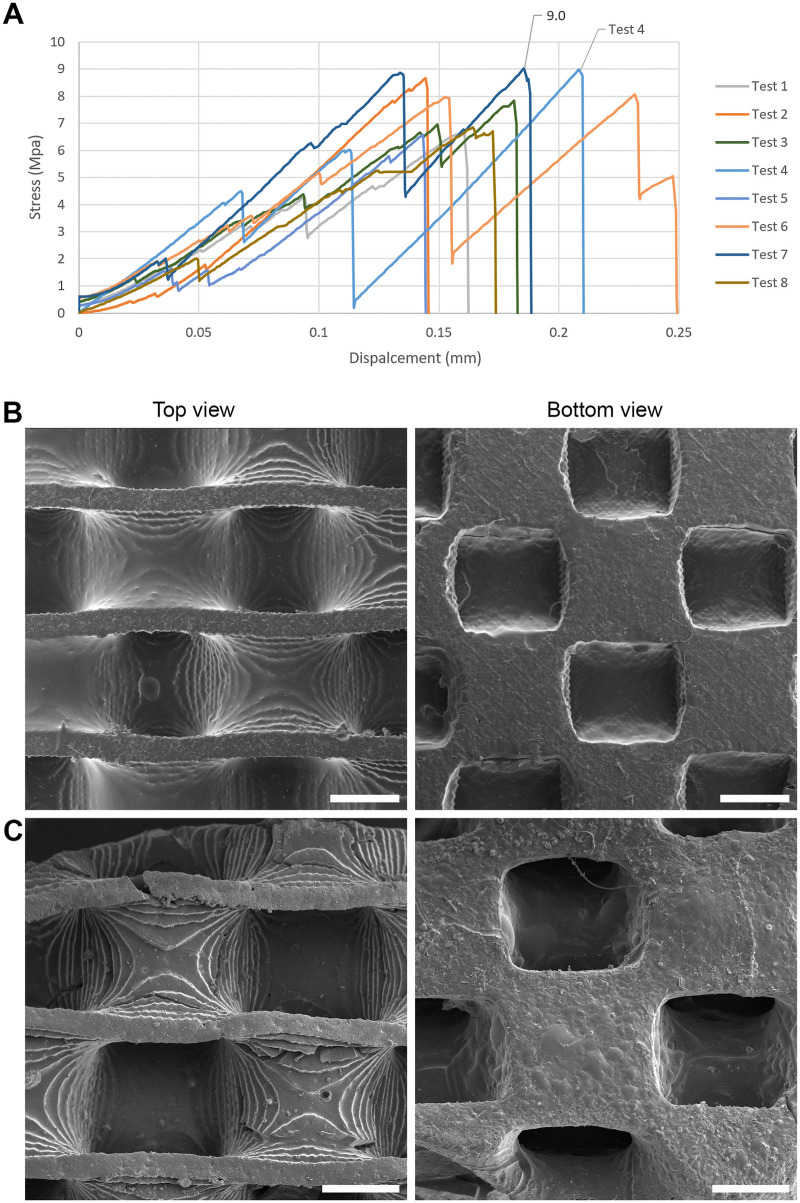
Mechanical and topographical analysis of SiOC scaffolds. **(A)** Stress x displacement curves obtained for eight bending tests. **(B)** SEM imaging of the top and bottom region of printed scaffolds. Scale bar (SB) = 1 mm. **(C)** SEM imaging of the top and bottom region SiOC pyrolyzed scaffolds. SB = 500 µm.

The tests exhibited similar behavior until they reached their first significant stress peaks (Figure 3A). Some specimens fractured completely, while others experienced abrupt drops in stress without complete fracture (damage tolerance behavior). An example of this behavior can be seen in the curves of test 4, where three significant stress drops occur, indicating fractures in the specimen. However, the final fracture only occurs after a displacement of 0.21 mm. A similar behavior was observed for test 6, where the displacement of the sample extends up to 0.25 mm, with two prominent stress peaks reaching 7.9 MPa and 8.1 MPa.

Overall, the minimum displacement for fracture was approximately 0.145 mm and occurred in test five, while the maximum stress obtained was 9.0 MPa and occurred in test 7.

### 3.3 Morphology and linear retraction

Analyzing the pattern of the base layer filling between the printing and pyrolysis stages (Figures 3B, C), it could be observed that the square pattern of the layer is maintained through uniform shrinkage of the piece. Regarding the size of the macrostructures, their average lengths reduced from ∼1.25 mm before pyrolysis to ∼0.73 mm after pyrolysis, indicating an average length reduction of approximately 51.33%.

As for the surface quality of the scaffolds, despite the high shrinkage, the samples exhibited a homogeneous pattern with few irregularities and “vesicle” characteristics, which may be associated with the release of gases from the polymers during the pyrolysis process. Some small fissures still appeared in edge regions, but not in sufficient quantity to cause significant damage to the pieces. The layer-by-layer photopolymerization process was visible (Figures 3B, C), primarily observed on non-flat surfaces (convex, concave) with a height dimension exceeding the printing layer height, set at 100 µm.

### 3.4 SiOC scaffolds are non-cytotoxic and allow cell adhesion and cell growth

**FIGURE 4:**
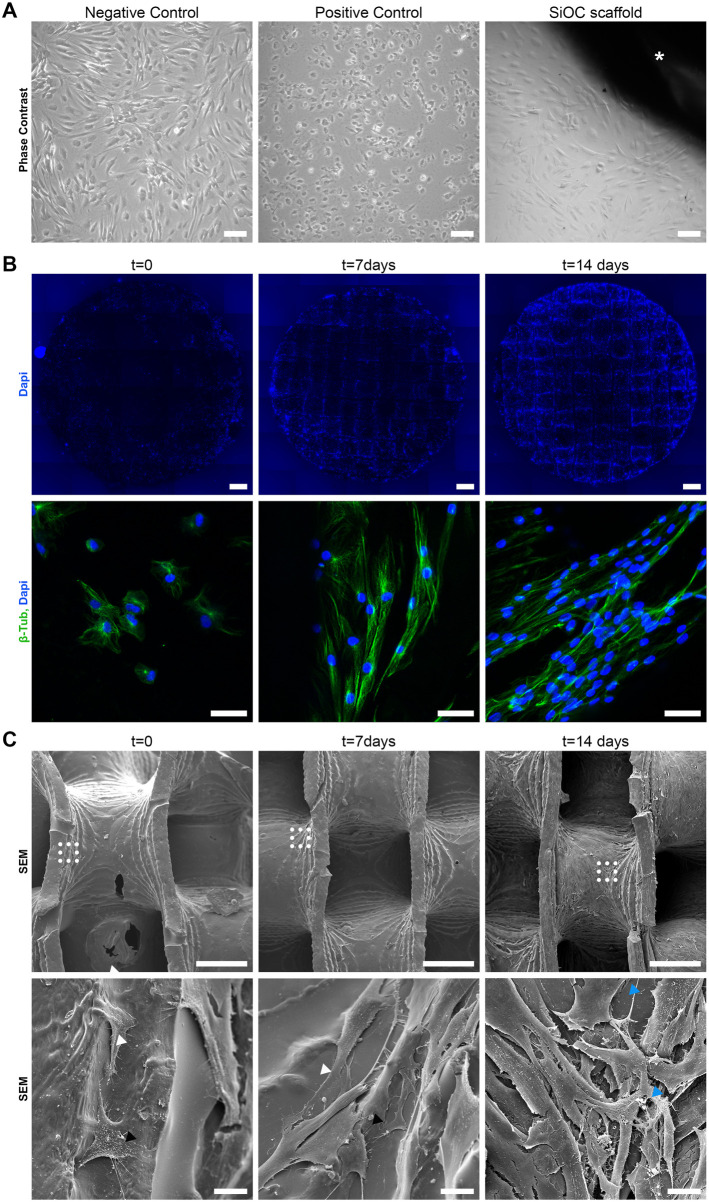
SiOC scaffolds are non-cytotoxic and allow adhesion, growth, and spreading of hASCs. **(A)** Representative images of direct contact assay demonstrate the morphology of hASCs kept in culture medium (negative control), treated with SDS (positive control), or kept in contact with SiOC scaffolds (*) for 48 h (n = 3 technical replicates). SB = 100 µm. **(B)** Widefield (top) and confocal (bottom) microscopy analysis of hASCs adhesion, growth, and spreading in SiOC scaffolds. Comparison of scaffolds immediately after cell seeding (t = 0) and after 7 and 14 days in culture (n = 1). SB = 1 mm and 50 µm in top and bottom, respectively; cell nuclei are stained with Dapi, and the cytoskeleton is labeled with an anti-β-Tubulin antibody. **(C)** SEM analysis of hASCs morphology after cell seeding in SiOC (t = 0) and after 7 and 14 days in culture (n = 1). The bottom panels show a higher magnification of dashed areas shown in the top panels. White arrohead = lamellipodia and filopodia; black arrowhead = lateral protrusion; blue arrowhead = fibrous deposition. SB = 500 µm (top) and 20 µm (bottom).

Next, we evaluated the biological performance of the SiOC scaffolds produced. Adult stem cells, such as hASCs, are one of the leading players in tissue regeneration. To evaluate the potential use in tissue engineering, we first accessed the cytotoxicity of the SiOC scaffolds on hASCs by a direct contact assay. We observed that hASCs in the negative control (Figure 4A, first panel) formed a homogeneous monolayer without any remarkable morphologic changes. In contrast, cells in the positive control (treated with SDS) were rounded and lost their monolayer conformation, suggesting a cell lysis process (Figure 4A, middle panel). Notably, cells in contact with the SiOC scaffolds (Figure 4A, last panel) maintained the fibroblastic morphology and monolayer organization as the negative control, suggesting that the scaffolds were not cytotoxic.

We followed the adhesion, spreading, and growth of hASCs on the scaffolds over time by microscopy (Figures 4B, C). At t = 0, it was possible to visualize the initial adhesion of hASCs after cell seeding, which were concentrated at the edges of the SiOC scaffold (Figure 4B, first column), possibly due to the seeding method. Moreover, β-tubulin staining showed at t = 0 (Figure 4B, first column) the initial morphology of the hASCs, with the cells still in a round shape, starting to adhere to the scaffold. SEM (Figure 4C, first column) showed in more detail the initial contact of hASCs with the SiOC surface, making it possible to visualize the filopodia, lamellipodia, and other membrane protrusions of the cells, representing the adhesion process and the activity of the cytoskeleton of hASCs.

At t = 7d, greater coverage of SiOC scaffold by hASCs was observed (Figure 4B, middle column), and the number of nuclei stained by DAPI demonstrated the presence of more cells than at the previous time. β-tubulin staining (Figure 4B, middle column) showed a greater spreading of the cytoskeleton, with a fibroblastic morphology, different from the t = 0. Similarly, SEM (Figure 4C, middle column) showed increased cell membrane adhesion to the SiOC scaffold, with the membrane protrusions in contact with the scaffold surface.

At t = 14d, the surface of SiOC was almost entirely covered by the hASCs (Figure 4B, last column). There was a significant increase in the number of cells in the scaffold’s central region, unlike at t = 0 when cells were mainly at the edges of the device. With a morphology like that of hASCs at 7 days, cells at t = 14 stained with anti-β-tubulin showed a spreading of the membrane but with an evident higher number of cells (Figure 4B, last column). At this point, the SEM (Figure 4C, last column) showed complete coverage of the 3D surface of the scaffold, forming a uniform cell monolayer. Remarkably, the cells still show filopodia and membrane protrusions, and deposits of fibrous extracellular matrix surrounded the cells (Figure 4C, last column).

**FIGURE 5:**
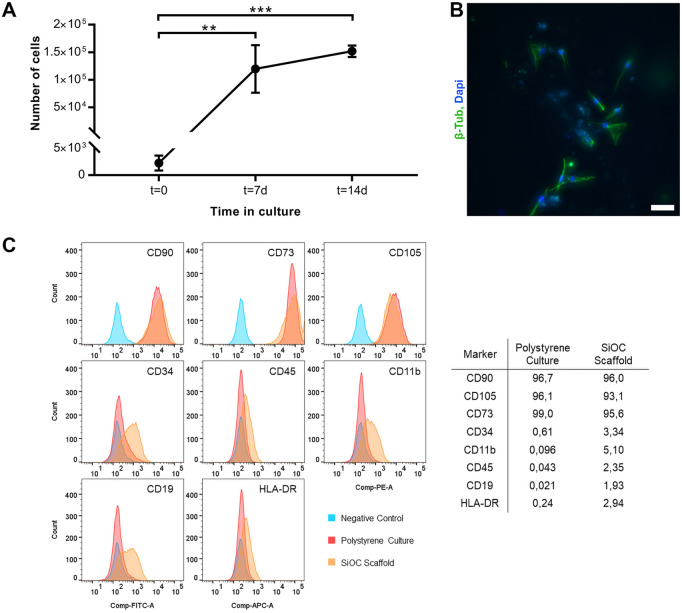
hASCs cultivated in SiOC scaffold maintain their phenotype. **(A)** Growth curve of hASCs cultivated in SiOC scaffold for up to 7 days (n = 3 independent experiments). Mean ± Standard deviation (SD); Shapiro-Wilk normality test; ANOVA with Tukey’s multiple comparisons test. **(B)** Colonization of SiOC scaffolds by hASCs. SiOC scaffolds were placed on top of hASCs grown in 2D polystyrene plates. After 2 days, we observed cells adhered to the surface of the 3D scaffold (n = 1). SB = 50 μm; cell nuclei are stained with Dapi, and the cytoskeleton is labeled with an anti-β-Tubulin antibody. **(C)** Immunophenotype of hASCs cultivated in SiOC scaffolds for 14 days (n = 1).

The increasing coverage of the scaffold surface by the hASCs is in accordance with the growth curve performed (Figure 5 A). At t = 0, there was an average of 2.25 × 10^3^ ± 1.4 × 10^3^ cells/scaffold, showing a low attachment rate of the cells (adhesion of 2.25% of the seeded cells). However, at t = 7, the cell count increased more than 50 times, reaching a value of 1.2 × 10^5^ ± 0.4 × 10^5^ cells/scaffold. At the last time point, t = 14, the number of hASCs was 1.5 × 10^5^ ± 0.1 × 10^5^ cells/scaffold, more than 60 times than at t = 0 and 1.25 times than at t = 7.

The results suggested that cells migrate and proliferate over the entire surface of the SiOC scaffolds. A colonization assay was carried out to check the capacity of the hASCs to migrate from a monolayer in a 2D culture plate to the SiOC scaffold. After 2 days, the device was removed from the culture plate and the hASCs on the scaffold were stained with anti-β-tubulin and DAPI (Figure 5B), showing that the cells could migrate and adhere to the SiOC surface.

In addition to cell adhesion and culture, the immunophenotype assay was performed to confirm the maintenance of stem cell marker patterns for a 14-day culture on SiOC. The assay showed that the analyzed surface markers remained unchanged in both types of hASCs culture - polystyrene culture flasks and SiOC culture (Figure 5C). For the positive markers, CD90, CD105, and CD73, the percentage of labeled cells remained above 90%, with 96%, 93.1%, and 95.6%, respectively for SiOC. The negative markers were up to 5%, with 3.34% for CD34, 5.10% for CD11b, 2.35% for CD45, 1.93% for CD19, and 2.94% for HLA-DR, for SiOC.

### 3.5 Bone-like matrix deposition in SiOC scaffolds

**FIGURE 6:**
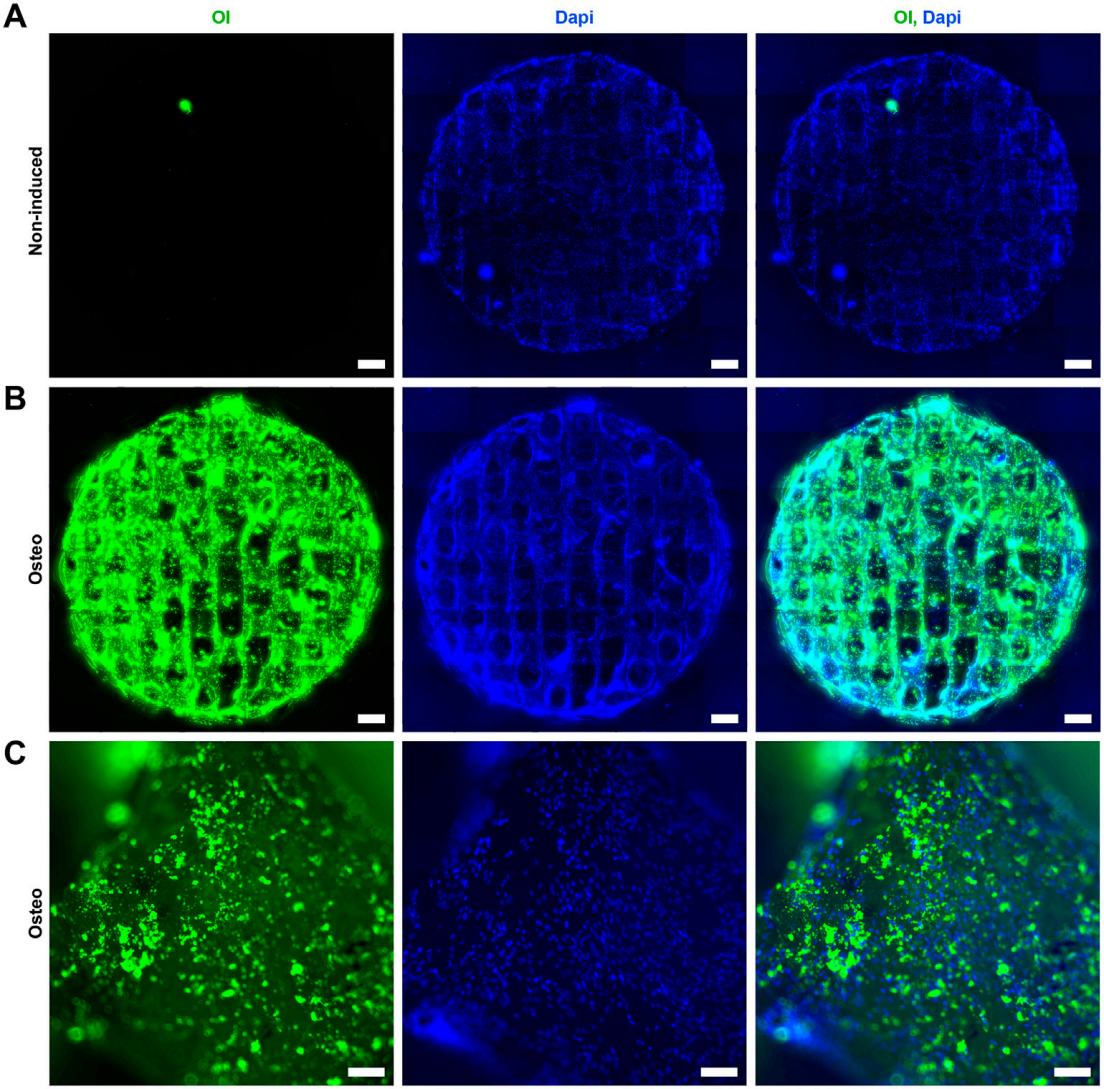
SiOC scaffolds are osteoconductive. hASCs were cultivated in SiOC scaffolds for 7 days and then treated with **(A)** non-inducing or **(B)** osteogenesis-inducing medium for 21 days (n = 3). The formation of an extracellular matrix rich in hydroxyapatite was observed using a hydroxyapatite fluorescent dye (OI). SB = 1 mm; cell nuclei are stained with Dapi. **(C)** Higher magnification of the hydroxyapatite deposits observed in the cells induced to osteogenesis. SB = 100 µm; hydroxyapatite deposits are stained with OI, and cell nuclei are stained with Dapi.

After confirming the cytocompatibility and the possibility of culturing hASCs on scaffolds without changing the main characteristics of the cells, we analyzed the osteoconductive potential of the SiOC scaffolds, that is, the ability to allow the bone to grow on its surface (Albrektsson and Johansson, 2001). The osteoblasts act in bone regeneration and homeostasis (Lin et al., 2020), synthesizing extracellular matrix rich in proteins and mineral deposition, such as hydroxyapatite. The differentiation of hASCs, cultured in SiOC and treated with osteogenic induction medium, in osteocytes was confirmed using OsteoImage Kit (Figures 6A–C), which stains the hydroxyapatite deposits released by the cells. The fluorescence microscopy provided evidence of the effectiveness of differentiation. In the non-induced control, only DAPI staining was visualized (Figure 6A), demonstrating that no hydroxyapatite deposition occurred, in contrast to the induced SiOC, for which OsteoImage staining was observed throughout the surface of the scaffold (Figure 6B). In more detail, extracellular matrix deposits were evidenced (Figure 6C). Moreover, there was a higher density of cells in the scaffold induced for osteogenesis, possibly due to increased cell compaction and/or cell organization in multiple layers (Figures 6A, B, middle panel).

**FIGURE 7:**
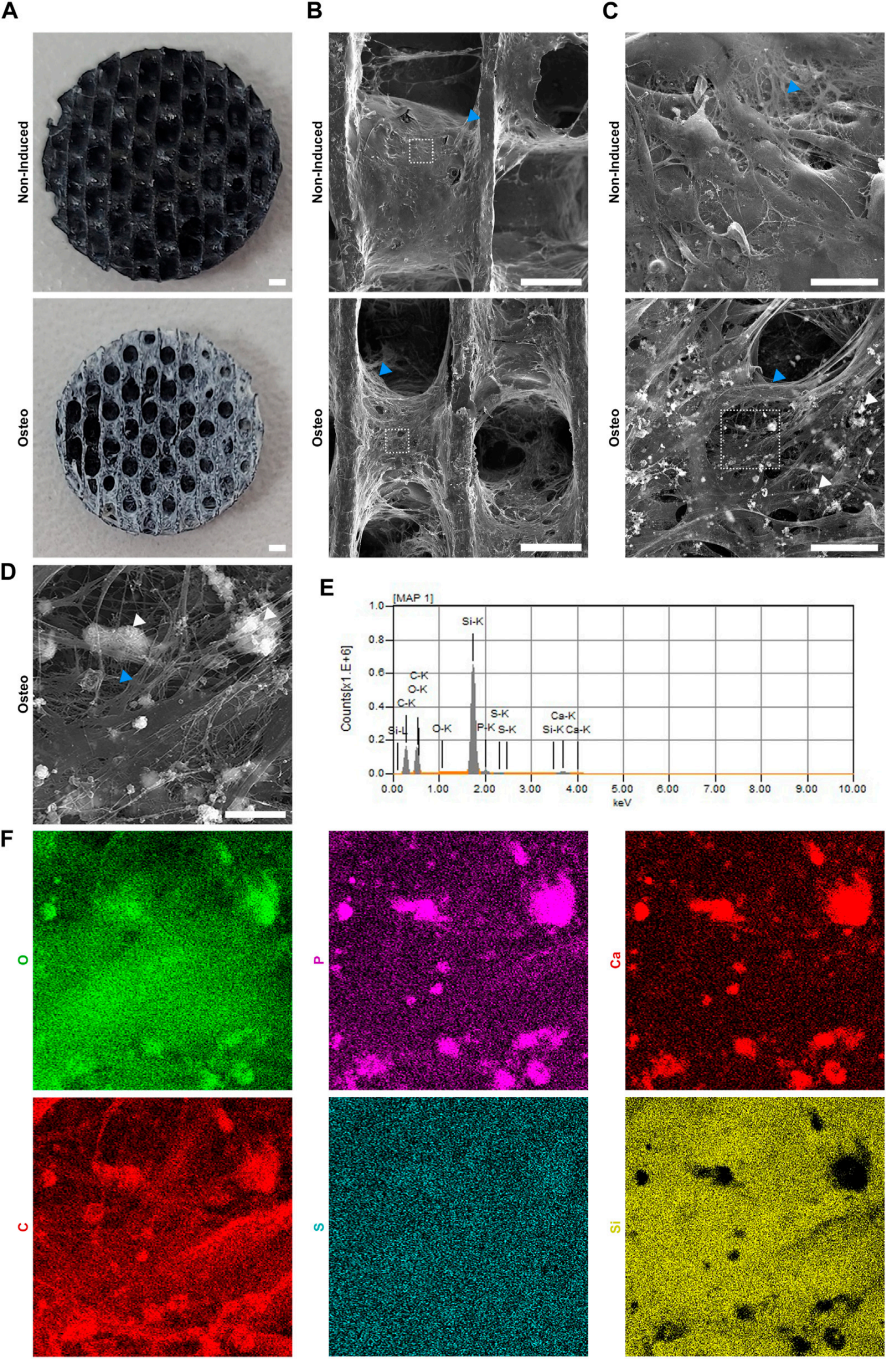
hASCs grown in SiOC scaffold and induced for osteogenesis secrete a fibrous matrix enriched in calcium phosphate deposits. **(A)** hASCs were cultivated in SiOC scaffolds for 7 days and then treated with a non-inducing (top) or osteogenic-inducing (bottom) medium for 21 days. After fixation and drying (by critical point), a dense whitish matrix was observed in the scaffolds induced to osteogenesis. SB = 1 mm. **(B)** SEM analysis of non-induced and osteogenesis-induced hASCs in SiOC scaffolds. SB = 500 µm. **(C)** Higher magnification of dashed areas shown in **(B)**. SB = 50 µm. **(D)** Higher magnification of the dashed area shown in the C bottom. SB = 10 µm. **(B–D)** White arrowhead = granular deposition; blue arrowhead = fibrous deposition. **(E)** Spectrum and **(F)** mapping obtained by EDS analysis of the region shown in **(D)**. O = oxygen; P = phosphorus; Ca = calcium; C = carbon; S = sulfur; Si = .silicon.

To further evaluate the extracellular matrix produced by the induced cells on the SiOC scaffolds, SEM analysis was performed. Notably, after fixing and drying (for SEM), it was observed that the induced scaffold, unlike the non-induced one, had a dense and whitish coating, possibly related to the deposition of the extracellular matrix (Figure 7A). SEM images demonstrated that the non-induced scaffold was covered by cells surrounded by a fibrous matrix (Figures 7B, C, top panels). In the induced SiOC, it was possible to observe the organization of cells in multiple layers, with the deposition of a rich fibrous matrix, forming a tissue-like structure, partially filling the pores of the scaffold. The matrix of the induced SiOC also had granular deposits (Figures 7B, C, bottom panel, and Figure 7D). EDS analysis (Figures 7E, F) corroborated that these granules were rich in phosphorus, oxygen, and calcium, components of hydroxyapatite. Moreover, the EDS analysis identified carbon, sulfur, and silicon, compatible with biological material and the SiOC surface. EDS analysis of non-induced cells did not show the presence of these granules containing calcium and phosphate (Supplementary Figures S2A–C).

## 4 Discussion

Bone grafts are still one of the main treatment strategies for treating critical-sized bone loss. The use of autografts is considered the gold-standard procedure since it contains bone matrix, biological cues, and cells to accelerate tissue regeneration but is limited by low yield and pain in the donor site (Brink, 2021). Using allogenic material may be hampered by immunological sensitization (reaching an average of 48% of the patients) (Moraschini et al., 2020) and the risk of pathogen transmission and infection, with studies reporting a rate of bacterial contamination in 2.6%–35% of the grafts (Brink, 2021). Another possibility is using a demineralized bone matrix (DBM) consisting of allogenic tissue that passes through a standardized decalcification process (on acid solution). This procedure removes living cells and reduces the risk of pathogen transmission but maintains the protein content, such as collagen and growth factors. DBM preserves an important osteoinductive activity but has low mechanical performance (Brink, 2021). As alternative, bone tissue engineering seeks to combine different biomaterials and manufacturing techniques to develop new tissue substitutes.

The use of SiOC-based materials has been explored due to their properties such as temperature resistance, mechanical strength (elastic modulus, bending strength, and hardness), chemical durability, and corrosion resistance (Lagonegro et al., 2017; Arango-Ospina et al., 2020). Here, we used DLP followed by pyrolysis to produce a 3D SiOC scaffold. Structures with complex architecture may be manufactured by DLP (Schmidt and Colombo, 2018), which can be applied to produce personalized tissue substitutes. Compared to other photopolymerization techniques such as stereolithography, the DLP is faster owing to the use of a projector and reduces the risk of printing defects due to the bottom-up printing system. Moreover, adding photosensitive pre-ceramic polymers substitutes ceramic particles and dispersants, which makes it challenging to maintain the viscosity of the resin (Schmidt and Colombo, 2018). On the other hand, some parameters still need to be optimized to use SiOC as a tissue substitute for bones, such as controlling the degradation rate, improving mechanical properties, and the ability to form apatite in contact with body fluids. The development of SiOC devices also opens doors for adjustments in topography and porosity, the association with bioactive elements such as metal ions (Xie et al., 2019; Arango-Ospina et al., 2020) or even optimized for applications such as drug delivery (Arango-Ospina et al., 2020).

The identification and evaluation of phases are essential factors in determining the properties of the studied ceramic material. In the literature, crystalline and amorphous bioactive glasses are found mainly dependent on their chemical composition and the thermal treatment employed during fabrication (Suttor et al., 1997; Esfehanian et al., 2008). For the material used in this project, XRD exhibited a peak around 2θ = 22°, which is a well-known XRD pattern for amorphous silica (Grishin and Smirnov, 2021), while the second, subtler peak observed around 2θ = 40° can be characterized as the XRD pattern of graphite carbon (Feng et al., 2015). Furthermore, no small peaks, as seen in the work of Schell et al. (Schell et al., 2010), are observed, indicating that the structure is exclusively amorphous, without nanoscale carbon precipitates or crystalline phases such as SiC or SiO_2_. These observed characteristics may favor the material’s structure, considering that crystalline precipitates in an amorphous matrix can generate stresses at the interface between the crystals and the matrix, leading to the initiation and propagation of microcracks, resulting in material failure.

The mass variation and geometric shrinkage analysis results by scanning electron microscopy (SEM) were similar to those obtained by N. Brodnik et al. (Brodnik et al., 2020), where SiOC-based ceramic scaffolds were also produced via DLP printing and pyrolysis. The authors evaluated the volumetric and linear shrinkage of the K cell, octahedral, and mixed filling patterns. They measured linear shrinkages ranging from 43.4% to 49.5% for the fabricated samples, with volumetric shrinkages reaching 78%. Electron microscopy observations revealed that all geometries exhibited excellent surface quality and isotropic shrinkage without deformation-related failures during the debinding process. In another study by Y. Zeng et al. (Zeng et al., 2018), DLP technology was employed to print simple thin-wall geometries in hydroxyapatite, resulting in good surface quality. Overall, geometric patterns and control of heating rates during the debinding process are the main factors in achieving isotropic structural shrinkage of the fabricated parts.

The type of technology used, and the composition of the raw material are crucial for the shrinkage behavior of the parts. In a study by Z. Liu et al. (Liu et al., 2019), using DLP technology but for the fabrication of hydroxyapatite (HAp) ceramic scaffolds, the measured volumetric shrinkage of the samples ranged around 72.8%. In the work by L. Diaz et al. (Diaz-Gomez et al., 2020), a composite of HAp and beta-tricalcium phosphate was extruded as a paste and sintered at high temperatures. The linear shrinkage obtained with the samples was minimal, around 5%. These results suggest that the produced scaffolds exhibit high shrinkage rates but are consistent with the technology employed for their production. An essential aspect to evaluate, therefore, is the isotropy of the observed shrinkage in the scaffolds. Such behavior is directly related to the type of cell pattern and the homogeneity of mass loss during the pyrolysis process.

The SiOC samples presented characteristic bands of the Si-O, Si-C, and Si-O-Si, as previously decribed (Zakirov et al., 2007; Su et al., 2010; Widjonarko et al., 2014; Lu et al., 2016a; Lu et al., 2016b; Wang et al., 2019; Ou et al., 2020; Alcaraz et al., 2021; Leonel et al., 2023; Niu et al., 2023). The pyrolyzed sample had a thermogravimetric behavior predicted for SiOC samples, with no change in mass up to 900 °C (Leonel et al., 2023). Above 1000°C, detoration of the material was observed, either by reacting with small quantities of free oxygen in the test chamber or by reacting with the recombination of the SiOC to form SiOx, COx or SiCx species (Lu et al.). For the resin sample, the weight loss behavior is very similar to what is found in the literature on TGA test of Tego RC711, Silres H44, and its mixtures, which is the case of this work (Colombo et al., 2010; Schmidt and Colombo, 2018; Huang et al., 2020). Our results also confirmed the SiOC scaffold’s ability to release Si at a constant rate in the first 10 days. Notably, other groups found a more pronounced Si release from silicon-based scaffolds the first 2 days of assay, which reducing subsequently (Yang et al., 2021; Yunsheng et al., 2023).

Obtaining sufficiently porous parts with mechanical performance close to that of cancellous bones is of interest in bone tissue engineering. An ideal bone tissue scaffold should have an interconnected and permeable porous structure, allowing tissue growth, nutrient distribution, and removal of metabolic waste and cellular products (Yang et al., 2004; Rezwan et al., 2006). Furthermore, the pore size also influences the ability of bone tissue to grow, with larger pore diameters (>500 µm) favoring osteogenesis due to enhanced vascularization (Karageorgiou and Kaplan, 2005). For the produced cylindrical scaffolds, the initial conditions of porosity and macropore size were achieved through a well-porous structure (72.7% ± 1.0%) with an interconnected and well-ordered chain of macropores, presenting sizes that promote the functionality of the artificial tissue (741.6 ± 39.6 µm). Regarding the manufacturing process control, the structures were designed with macropore diameters of 1.5 mm, while the printed pieces exhibited diameters around 1.43 ± 0.06 mm, indicating a percentage difference of 4.9%. This process precision is comparable to the ceramic slurries manufactured via DLP (Zeng et al., 2018; Li et al., 2021). Additionally, a density of 1.92 ± 0.02 g/cm³ was determined for the produced SiOC during the porosity determination process, which falls within the range of 1.5–2.5 g/cm³ reported in the literature for the material (Kim et al., 2003).

As introduced earlier in the literature review, there is an inversely proportional relationship between macropore size/porosity and the mechanical strength of the produced parts. However, factors such as raw material, manufacturing technology, and process parameters, among others, still influence the mechanical performance of the part. The literature on bioactive glasses produced via additive manufacturing shows that these materials can exhibit high porosity while maintaining good mechanical integrity. For example, in the study by Adam Marsh et al. (Marsh et al., 2021), the mechanical and porosity characteristics of silicate-based scaffolds produced via Fused Filament Fabrication (FFF) extrusion were evaluated. The porosity found for the parts after pyrolysis was 70% ± 4.9%, with an average pore size of 622 ± 139 µm and a compressive strength of 2.8 MPa. Similar articles report similar behaviors (Eqtesadi et al., 2014; Fiocco et al., 2017; Li et al., 2021), where highly porous structures (60%–80%) with interconnected macropore chains of various sizes exhibited compressive strengths within the range of 2–12 MPa, which aligns with the strength limits for cancellous bones (Hench, 1998).

The flexural strength of 7.8 ± 1.0 MPa obtained for the SiOC scaffolds was higher compared to previous studies involving ceramics for tissue engineering. X. Hockin et al. (Xu et al., 2004) reported a maximum flexural strength of 3.3 ± 0.4 MPa for calcium phosphate samples, while the article by Q. Chen and Boccaccini (Chen and Boccaccini, 2006) addressed PDLLA-coated bioactive glasses in their structure, reporting flexural strengths of up to 1.5 MPa. Additionally, the distinct fracture behaviors observed in the three-point flexural tests can be justified by the complex internal architecture of macropores and the density of defects present in each piece, leading to varied crack propagation and fracture behaviors. Thus, progressive cell fractures can be observed, associated with a material exhibiting damage-tolerant behavior.

When compared to the properties of cancellous and cortical bones, it is known that flexural strength can vary due to various factors such as age, gender, bone density, and anatomical location (Pastor et al., 2022). Within this context, the study by F. Pastor et al. evaluated bones of canids (*Cerdocyon thous*) through three-point bending tests. The authors found that the average flexural strength for the humerus was 7.4 MPa, while for the femur, it was 5.5 MPa. Additionally, bone length, weight, and width influence mechanical behavior (Pastor et al., 2022). In the article by J. Lotz et al., a quantitative study on the mechanical properties of human cancellous bones was conducted, indicating estimated strengths of approximately 15 MPa (Lotz et al., 1990). Another reference states that human cancellous bone varies in its maximum strength between 1.5 and 38 MPa (Liu et al., 2007). These results suggest that the flexural modulus obtained for the ceramic scaffolds manufactured for this article meets the mechanical expectations for applications in tissue engineering.

Finally, the average hardness of 935.1 ± 71.0 HV obtained fell within the reported hardness values for SiOC in the literature. The literature indicates a hardness range of 8–9 GPa in the study by R. Doremus (Doremus and Prochazka, 1991) and a range of 7.4–7.8 GPa in the work of G. Domenico et al. (Sorarù et al., 2020). These ranges correspond to Vickers hardness values of 700–1000 HV.

One of the goals of using bioactive glasses as bone substitutes is to fulfill not only the role of support and protection but also stimulate tissue regeneration. Bone regeneration is known to occur by recruiting MSCs to the injury site. This is followed by cell proliferation, osteoblastic differentiation, matrix deposition and intramembranous ossification (Lin et al., 2020; Meesuk et al., 2022). Here, we performed *in vitro* tests to assess the potential of SiOC scaffolds to support adhesion, growth, and colonization by adult stem cells.

The scaffold must be biocompatible to be used as a tissue substitute (Tang et al., 2008). Following ISO 10993, a series of tests must be performed to prove the biocompatibility of a biomaterial: cytotoxicity, sensitization, irritation, acute systemic, subacute and chronic toxicity, genotoxicity, immunoresponsiveness, hemocompatibility carcinogenicity, degradation and implantation (Huzum et al., 2021). Here, we performed the first analysis of this flowchart in order to show that the SiOC scaffolds were not cytotoxic and allowed cell adhesion and growth. Cytocompatibility might be influenced, among other parameters, by the production process of the biomaterial. Mesoporous SiOC particles were not cytotoxic to lymphoblastic and uterus/endometrium epithelial cell lines (Tamayo et al., 2015). Carbon-dopped silica nanowires were also not cytotoxic and supported the proliferation of murine fibroblasts (Lagonegro et al., 2017). Porous SiOC scaffolds produced by using thermoplastic polyurethane and impregnated with pre-ceramic polymer polysilazane and pyrolyzed were biocompatible with embryonic murine fibroblasts, osteosarcoma cells (Kulkarni et al., 2021) and human bone-marrow-derived mesenchymal stem cells (Yang et al., 2021). Layers of black glasses (SiOC-based) on titanium substrates showed no cytotoxic effect on osteosarcoma cells (Gawęda et al., 2018). Ca- and Mg-modified silicon oxycarbide glasses prepared from a polymeric single-source precursor were also compatible with mouse embryonic fibroblasts and human embryonic kidney cells (Gonzalo-Juan et al., 2016). The results obtained in the present work followed these previous studies and demonstrated that non-cytotoxic SiOC scaffolds can be produced from PDC using DLP and pyrolysis.

After cell seeding on the scaffolds, we obtained a low rate of initial adhesion, and the cells were concentrated on the edges of the scaffolds. This may be related to the process of cell seeding, which may favor the contact of the cells in suspension with the lateral area of the scaffold. Furthermore, the scaffold surface was partially hydrophobic (data not shown), which may have hindered this initial contact. These problems may be overcomed by using different seeding technics (Olivares and Lacroix, 2012; Buizer et al., 2014) or surface treatments to improve hydrophilicity (Langowski and Uhrich, 2005; Ghezzi et al., 2021). Nevertheless, after 7 days in culture, the cells recovered the number of hASCs seeded, demonstrating the potential of these cells to populate the SiOC scaffold. On the other hand, the lower cell growth between days 7 and 14 may be caused by the cell-cell inhibition due to the higher scaffold coverage, as was observed by microscopy.

The visualization of lamellipodia and filopodia demonstrated the interaction between the cells and the SiOC surface and the migration and anchorage of the hASCs on the culture conditions (Collart-Dutilleul et al., 2014). The emission of thin membrane protrusions is related to the migration and proliferation process and also indicates how the cell senses the surrounding surface (Collart-Dutilleul et al., 2014). We also found that after 14 days of culture on SiOC, the hASCs kept the mesenchymal stem cell immunophenotype (Dominici et al., 2006), suggesting that the scaffold does not trigger the differentiation process. These cells have an critical paracrine activity for tissue regeneration, secreting immunomodulatory and pro-regenerative factors (Liu et al., 2021; Ivanisova et al., 2023).

Our results also demonstrated that, upon receiving chemical stimulation (inducing medium), the hASCs cultured in SiOC differentiated in osteoblasts. Bone tissue is characterized by an abundant extracellular matrix synthesized mainly by osteoblasts, composed of proteins, polysaccharides and mineral deposits (Lin et al., 2020). This composition is responsible for conferring the characteristic rigidity of bone tissue, which is directly related to the bone support and protection role. In addition, bone tissue also functions as a mineral reservoir, such as calcium phosphate (Peacock, 2021; Wawrzyniak and Balawender, 2022). The osteoblasts obtained in SiOC formed a dense fibrous extracellular matrix rich in hydroxyapatite. In addition, we observed the formation of a tissue-like layer on the SiOC surface in the differentiated hASCs, with a high density of cells, in agreement with other results of osteogenic differentiation *in vitro* (Meesuk et al., 2022).

## 5 Conclusion

SiOC scaffolds were produced using DLP followed by pyrolysis. The structures produced had an amorphous organization and a porous macrostructure with diamond TPMS cells. The scaffolds produced had mechanical properties compatible with bone tissue and did not show cytotoxicity in vitro assays. The SiOC scaffolds also functioned as a suitable surface for the adhesion and growth of adult stem cells, one of the leading players in tissue regeneration. Cells grown on these scaffolds could differentiate into osteoblasts, forming a bone tissue-like organization, with the secretion of a fibrous extracellular matrix rich in hydroxyapatite. The extracellular matrix is a fundamental component of bone tissue and is responsible for providing its rigidity and characteristic mechanical properties. We demonstrated that our SiOC scaffolds have potential for use in the treatment of critically sized bone lesions, presenting mechanical characteristics compatible with the use as a tissue substitute, but also pro-regenerative potential to stimulate bone tissue regeneration. More studies are still needed to confirm the compatibility of SiOC scaffolds with other elements of the tissue regeneration process, such as immune system cells, in addition to preclinical studies to evaluate *in vivo* the safety, compatibility and efficiency of this device.

## Data Availability

The original contributions presented in the study are included in the article/Supplementary Material, further inquiries can be directed to the corresponding authors.
