# Supplementary material for: Cytocompatible and osteoconductive silicon oxycarbide glass scaffolds 3D printed by DLP: a potential material for bone tissue regeneration
Source: Front Bioeng Biotechnol. 2024 Jan 4;11:1297327. doi: 10.3389/fbioe.2023.1297327 (PMC10794595; doi:10.3389/fbioe.2023.1297327)
Supplement: Supplementary file 1 [file DataSheet1.pdf]

Cytocompatible and Osteoconductive Silicon Oxycarbide Glass Scaffolds 3D Printed by DLP: A Potential Material for Bone Tissue Regeneration

Matheus Versão Carnieri[1,†], Daniele Freitas Garcia[2,†], Rafael Voltolini[1], Neri Volpato[1], Marcio Mafra[1], Euclides Alexandre Bernardelli[1], Marco Stimamiglio[2], Carmem Rebellato[3], Alejandro Correa[2], Lucas Freitas Berti[1,*], Bruna Hilzendeger Marcon[2,4,*]

SUPPLEMENTARY FIGURES

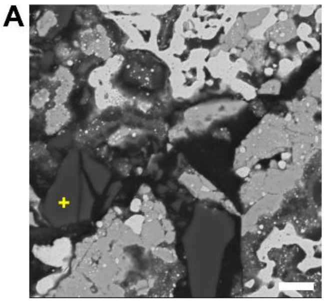

B

Formula	Mass (%)
C	27.83
O	53.02
Si	19.15
Cr	0.13
Fe	0.37

Figure S1. EDS point analysis of polished scaffolds. (A) MEV image showing point analysis (SB=10 µm) and (B) table with the elements found.

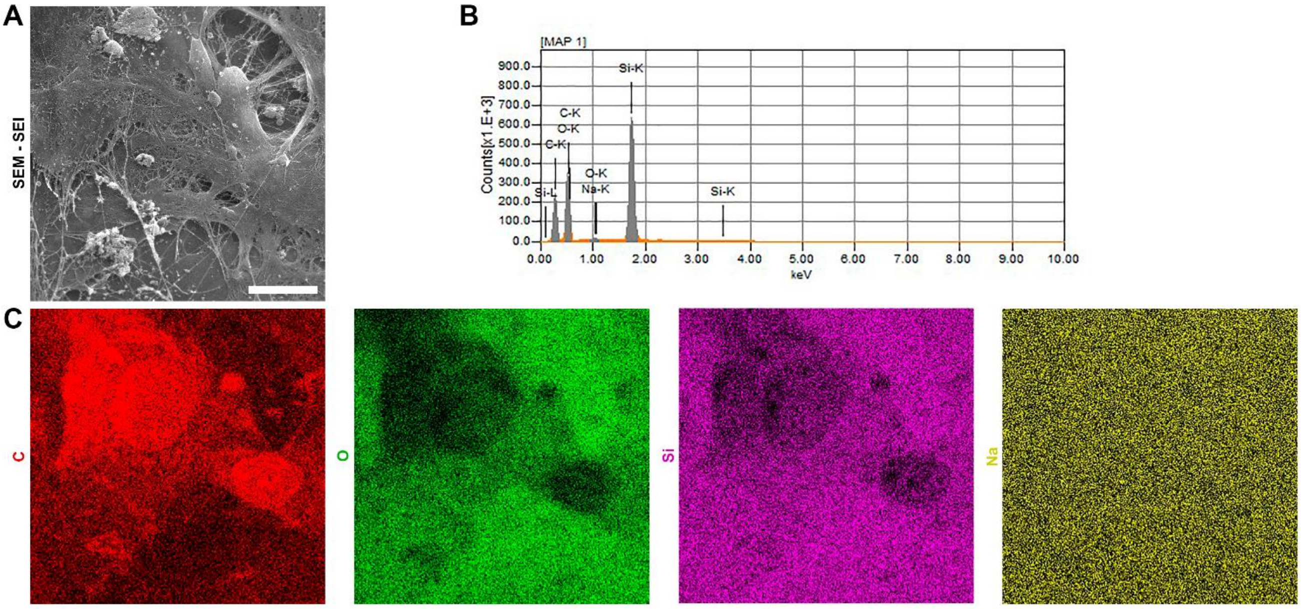

Figure S2. hASCs grown in SiOC for 28 days (non-induced). (B) SEM analysis of non-induced hASCs in SiOC scaffolds. SB=10 µm. (B) Spectrum and (C) mapping obtained by EDS analysis of the region shown in A. C=carbon; O=oxygen; Si=silicon; Na=sodium.
